# Assessment of Mortality Rate, Need for ICU Admission and Ventilation in COVID-19 Patients with Diabetes Mellitus

**DOI:** 10.5339/qmj.2022.9

**Published:** 2022-03-01

**Authors:** Mehrnoosh Zakerkish, Maryam Shaabanpour Fooladi, Hajieh Bibi Shahbazian, Fatemeh Ahmadi, Seyed Peyman Payami, Mehrdad Dargahi-Malamir

**Affiliations:** ^1^Diabetes research center, Health research institute, Ahvaz Jundishapur University of Medical Sciences, Ahvaz, Iran E-mail: Zakerkishm@yahoo.com; ^2^Department of Infectious Diseases, School of Medicine, Ahvaz Jundishapur University of Medical Sciences, Ahvaz, Iran; ^3^Department of Internal Medicine, School of Medicine, Ahvaz Jundishapur University of Medical Sciences, Ahvaz, Iran

**Keywords:** COVID-19, diabetes, death, ICU, invasive ventilation

## Abstract

Introduction: Coronavirus disease 2019 (COVID-19) has become a threat to public health. People with chronic diseases, such as diabetes, are at a greater risk of severe diseases and death upon contracting this new disease. Due to the novelty of COVID-19, no specific information is available about the degree of its mortality and risk factors among diabetic patients. Therefore, this study aims to compare diabetic and nondiabetic COVID-19 patients regarding mortality rate, the need for intensive care unit (ICU) admission, invasive and noninvasive ventilation, and the associated risk factors.

Methods: This was a cross-sectional study performed on the medical records of 650 adult COVID-19 patients (325 diabetics and 325 nondiabetics) admitted to Razi Hospital in Ahvaz from March 2020 to September 2020.

Results: The mean age of the patients was 61.3 years in the diabetic group and 52.3 years in the nondiabetic group. Men comprised 48.3% of the diabetic group and 59.7% of the nondiabetic group. Diabetic patients suffered from significantly more underlying diseases, such as ischemic heart disease (IHD), hypertension (HTN), chronic kidney disease (CKD), and acute renal failure (ARF) compared to the nondiabetic group (*p* < 0.0001). Also, when compared with the nondiabetic group, the diabetic group had a significantly higher mortality rate (17.5% vs. 12%; *p* = 0.047, respectively), more ICU admissions (35.4% vs. 27.7%; *p* = 0.035, respectively), and a greater need for invasive ventilation (17.5% vs. 11.4%; *p* = 0.026, respectively).

Conclusion: In diabetic patients, the mortality rate, need for ICU admission, and need for invasive ventilation were significantly higher than nondiabetic patients. Our logistic regression analysis in diabetic patients with COVID-19 showed that age, CKD, and ARF were the risk factors affecting mortality. In contrast, age and CKD were the risk factors affecting the rate of ICU admission, and CKD and ARF were the risk factors affecting the need for invasive ventilation.

## Introduction

Coronavirus is a ribonucleic acid (RNA) virus that has spread worldwide since the start of 2020.^1^ Although most infections with the virus are mild, what creates concern and worry about this virus is the high prevalence of two beta-coronaviruses that cause deadly pneumonia, including severe acute respiratory syndrome coronavirus-coronavirus 2 (SARS-COV), which became common in 2002–2003 and had a mortality rate of 10%, and Middle East respiratory syndrome coronavirus (MERS-COV), which was prevalent in 2012 and had a mortality rate of 36%.^2^ In December 2019, many cases of pneumonia of unknown etiology were reported in Wuhan City, Hubei Province, China. Studies of lower respiratory tract samples indicated a new coronavirus, which later became known as severe acute respiratory syndrome-coronavirus 2 (SARS-COV-2), and the resulting disease was coronavirus disease 2019 (COVID-19).^1–3^ This new coronavirus had higher transmissibility and lower mortality compared with SARS-COV.^4^

Diabetes is one of the causes of mortality and morbidity worldwide. Because of its association with macrovascular and microvascular complications, it affects patient survival.^5,6^ The link between diabetes and infection is well known in the literature.^7^ Infections, especially the flu and pneumonia, are more common and severe in elderly patients with type 2 diabetes.^8,9^ In this context, however, it remains controversial whether diabetes increases patients’ susceptibility to infection and affects the outcome of the infection or whether cardiovascular and renal comorbidities associated with diabetes are the major factors.^10^ Uncontrolled diabetes and hyperglycemia have been reported as predictors of disease severity and death in patients infected with various viruses, such as the influenza A pandemic in 2009 (H1N1), SARS-COV, and MERS-COV.^11,12^ COVID-19 is a current threat to public health, and people with chronic diseases, including diabetes, are at a greater risk for severe illness and death if they contract it.^13^ Meanwhile, since this disease is new, no published reports about its mortality rate and risk factors among diabetic patients are available. Therefore, the present study investigated the mortality rate, the need for intensive care unit (ICU) admission, invasive and noninvasive ventilation (NIV), and the associated risk factors among diabetic and nondiabetic patients.

## Methods

This was a cross-sectional study conducted using the medical records of adult COVID-19 patients admitted to Razi Hospital in Ahvaz, southwest of Iran, from March 2020 to September 2020. Inclusion criteria were adult patients with COVID-19, and the exclusion criterion was incomplete data obtained from medical records. This included missing data about past medical history, drug history, and other information that would prevent us from extracting study variables. A nonprobability and convenience sampling method was used for data collection, and all patients who met the inclusion criteria were included in the study. Sampling was continued until reaching the desired number of samples. The diagnosis of COVID-19 in patients was based on positive RT-PCR (Reverse Transcription Polymerase Chain Reaction) test results of throat and nasal swab specimens, clinical imaging manifestations, or Chest Computed Tomography scans indicating coronavirus pneumonia, as confirmed by the treating physician. The patient data were obtained from history forms, clinical charts, nurse notes, laboratory data, and physician reports, which were entered in a prearranged form. All patients had a unique national code, so there were no duplicate data. The study variables included: age, sex, diabetes (history of known diabetes or taking antidiabetic drugs), high blood pressure (history of high blood pressure or taking antihypertensive drugs), renal failure (history of chronic kidney disease (CKD) or being on dialysis,) and the incidence of acute renal failure (ARF); defined as an increase in serum creatinine of at least 0.3 mg/dl or an increase in serum creatinine 50% above baseline over the past 48 hours), cardiovascular diseases (history of cardiovascular diseases, including history of ischemic heart disease (IHD), history of ischemic cerebrovascular disease, and history of congestive heart failure; new incidence of cardiovascular diseases, including new incidence of acute coronary syndromes, cerebrovascular events, and congestive heart failure during a recent hospitalization), history of lung disease, history of cancer, use of corticosteroids and other immunosuppressants (oral or injectable drugs related to this class of drugs), mortality, the need for ICU admission, and the use of invasive and NIV.

The data collection tool included a checklist on which the variables were recorded. Based on the formula for calculating the sample size to compare the two populations, the diabetic and nondiabetic groups, with an alpha of 0.05 and a beta of 0.2, the sample size was determined to be 325 people for each group. A Chi-square test was used to compare the ratio in the two groups. An independent *t*-test was used to compare the mean of normal quantitative variables. *P* values < 0.05 were considered statistically significant. Data were analyzed using SPSS version 25, and logistic regression was used to determine the contributing risk factors. Data collection started after receiving approval from the Ethics Committee of Ahvaz Jundishapur University of Medical Sciences (Reference ID: IR.AJUMS.REC.1399.502).

## Results

**Figure 1.**
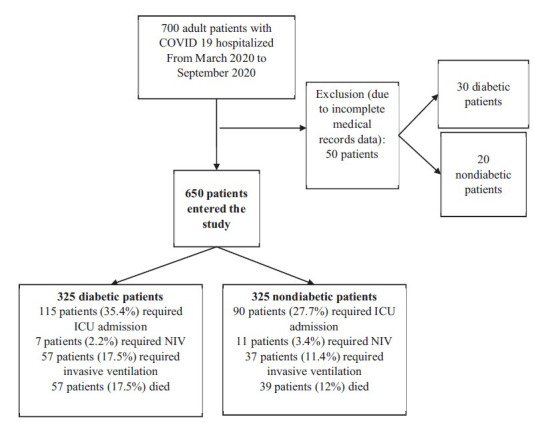
Flowchart of the study design.

The medical records data of 650 adult patients with COVID-19 in two equal groups of diabetics and nondiabetics (325 each) were reviewed (Figure 1). The mean age in the diabetic and nondiabetic groups was 61.3 years and 52.3 years, respectively, and this difference was significant (*p* < 0.0001). Men comprised 48.3% of the diabetic group and 59.7% of the nondiabetic group. The mean duration of diabetes in the diabetic group was 8.5 ± 6.5 years, with a minimum of one and a maximum of 20 years. Diabetic patients suffered from significantly more underlying diseases such as IHD, hypertension (HTN), CKD, and ARF compared with the nondiabetic group (*p* < 0.0001). Also, the diabetic group had a significantly higher mortality rate than the nondiabetic group (17.5% vs. 12%, respectively; *p* = 0.047). Regarding the need for ICU hospitalization, diabetic patients had a significantly higher need for ICU hospitalization than nondiabetic patients (35.4% vs. 27.7%, respectively; *p* = 0.035). A comparison of the need for invasive ventilation in diabetic and nondiabetic groups showed that 17.5% of diabetic patients required invasive ventilation. In contrast, this rate was 11.4% in nondiabetic patients, and this difference was statistically significant (*p* = 0.026). (Table 1).

Examination of the association of age with mortality, the need for ICU admission, and the use of invasive and NIV in diabetic and nondiabetic patients showed that in each age group, there was no significant difference between the death ratio in these two groups. However, in a separate analysis of these patients, the distribution of death in nondiabetic individuals in different age groups was significant. The highest mortality was in the age group ≥ 80 years, with 25% mortality (*p* = 0.032). The distribution of death in diabetics in different age groups was also statistically significant, and the highest death rate was in the age group of 70–79 years with 33.3% mortality (*p* = 0.007). The ratio of the need for ICU admission in the diabetic and nondiabetic groups showed a significant difference only in the age group of 40–49 years (36.4% vs. 14.5%) (*p* = 0.018). The distribution of the need for ICU admission in nondiabetic individuals in different age groups was significant, and the highest ICU admission was observed in the age group ≥ 80 years with a mortality rate of 53.6% (*p* = 0.003). The distribution of the need for ICU admission in diabetics in different age groups was also significant, and the highest ICU admission was seen in the age group of 70–79 years with a mortality rate of 54.4% (*p* = 0.037). Regarding the need for invasive ventilation in the age group of 40–49 years, the ratio of the need for invasive ventilation in the diabetic group was nearly four times that of the nondiabetic group (21.3% vs. 5.5%, respectively) (*p* = 0.036). There was no statistically significant difference in other age groups. In each age group, there was no significant difference between the ratio of the need for NIV in the diabetic and nondiabetic groups, except for the age group of 70–79 years (Table 2).

Comparing mortality, the need for ICU, and the need for invasive ventilation based on gender, there was no statistically significant relationship between women in the diabetic and nondiabetic groups (*p* value: 0.22, 0.16, and 0.13, respectively) and between men in these two groups (*p* value: 0.08, 0.057, and 0.058, respectively). There was no significant difference between women in diabetic and nondiabetic groups regarding the need for NIV (*p* = 0.73). However, this difference was significant between diabetic and nondiabetic men, with more nondiabetic men requiring NIV (0.6 vs. 4.1%, respectively; *p* = 0.046). There was no statistically significant difference in mortality, the need for ICU admission, and the need for invasive ventilation between nondiabetic men and women (*p* value: 0.54, 0.18 vs. 0.3, respectively). Also, there was no significant difference between diabetic men and women in this regard (*p* value: 0.31, 0.084 vs. 0.19, respectively).

The risk factors affecting mortality, the need for ICU admission, and the need for invasive ventilation in all patients were determined by entering the following variables into the logistic regression model: age, sex, history of diabetes, history of hypertension, CKD, the incidence of ARF, history of cardiovascular disease, the new incidence of cardiovascular diseases, history of lung diseases and cancer, and taking immunosuppressive drugs.

Age (OR: 1.038, 95% CI: 1.023–1.054, *p* < 0.0001), diabetes (OR: 1.560, 95% CI: 1.004–2.422, *p* = 0.048), history of CKD (OR: 2.846, 95% CI: 1.679–4.824, *p* < 0.0001), incidence of ARF (OR: 2.402, 95% CI: 1.529–3.772, *p* ≤ 0.0001), and cancer (OR: 3.267, 95% CI: 1.345–7.932, *p* = 0.009) had a significant association with mortality, and the highest risk was related to cancer (OR = 3.267).

Age (OR: 1.025, 95% CI: 1.014–1.036, *p* < 0.0001), diabetes (OR: 1.430, 95% CI: 1.025–1.994, *p* = 0.035), history of CKD (OR: 0.476, 95% CI: 0.300–0.754, *p* = 0.002), incidence of ARF (OR: 1.860, 95% CI: 1.290–2.682, *p* = 0.001), history of cardiovascular disease (OR: 1.768, 95% CI: 1.208–2.588, *p* = 0.003) and new incidence of cardiovascular disease (OR: 3.798, 95% CI: 1.759–8.198, *p* = 0.001) had a statistically significant relationship with ICU admission. The highest risk was related to new incidence of cardiovascular disease (OR = 3.798).

Diabetes (OR: 1.656, 95% CI: 1.060–2.586, *p* = 0.027), history of CKD (OR: 2.164, 95% CI: 1.126–4.157, *p* = 0.021), incidence of ARF (OR: 1.837, 95% CI: 1.019–3.311, *p* = 0.043), and cancer (OR: 2.716, 95% CI: 1.086–6.791, *p* = 0.033) had a significant association with the need for invasive ventilation. The highest risk was related to cancer (with OR = 2.71) (Table 3).

The risk factors affecting mortality, the need for ICU admission, and the need for invasive ventilation in diabetic patients were determined by entering the following variables into the logistic regression model: age, sex, history of hypertension, CKD, ARF, history of cardiovascular disease, the new incidence of cardiovascular disease, history of lung diseases, history of cancer, and the use of immunosuppressive drugs. Age (OR: 1.041, 95% CI: 1.016–1.067, *p* = 0.001), history of CKD (OR: 2.164, 95% CI: 1.126–4.157, *p* = 0.021), and incidence of ARF (OR: 2.010, 95% CI: 1.118–3.617, *p* = 0.02) had a statistically significant association with mortality in diabetic patients with COVID–19. The highest risk was related to a history of CKD (OR = 2.164). Age (OR: 1.020, 95% CI: 1.001–1.039, *p* = 0.036) and a history of CKD (OR: 1.876, 95% CI: 1.067–3.299, *p* = 0.029) had a significant relationship with ICU admission rate in diabetic patients with COVID-19. Also, the highest risk was related to a history of CKD (OR = 1.876). CKD (OR: 2.164, 95% CI: 1.126–4.157, *p* = 0.021) and incidence of ARF (OR: 1.837, 95% CI: 1.019–3.311, *p* = 0.043) had a significant relationship with the need for invasive ventilation in diabetic patients. The highest risk was related to CKD (OR = 2.164) (Table 4).

## Discussion

According to the literature, the prevalence of diabetes is 9.8% among patients admitted with COVID-19, while the prevalence of this chronic disease is 8.5% among the general population.^13^ Diabetic patients with other underlying diseases are more prone to ICU admission and mortality complications than patients of the same age and sex but without diabetes^13^. Several factors such as hyperglycemia, changes in cytokine production, impaired T cell-mediated immune response, inhibition of neutrophil chemotaxis, ineffective microbial clearance, and phagocytic cell dysfunction may exacerbate immune dysfunction in diabetics.^14^ ACE2 may play a vital role in the severity of COVID-19 infection in diabetics because it is expressed in pancreatic tissue, and the virus uses this enzyme to attack host pneumocytes. In addition, proinflammatory cytokine levels, particularly IL-1, IL-6, tumor necrosis factor α (TNF-α), and various markers, such as CRP, D-dimer, and fibrinogen, may increase cytokine storms and lead to severe disease in diabetics with COVID-19 infection.^15,16^

Compared to nondiabetic patients, the mortality rate, the need for ICU admission, and invasive ventilation were significantly higher in diabetic patients in this study. Based on logistic regression analysis of risk factors affecting mortality, the need for ICU admission and invasive ventilation in the entire study population, age, diabetes, history of CKD, the incidence of ARF, and cancer were the factors affecting mortality. Factors such as age, diabetes, history of CKD, the incidence of ARF, history of cardiovascular disease, and recurrence of cardiovascular disease were affecting ICU admission. Finally, factors, including diabetes, cancer, history of CKD, and ARF incidence, contributed to the need for invasive ventilation. Using a logistic regression model, the evaluation of risk factors affecting mortality, the need for ICU admission, and invasive ventilation in diabetic patients with COVID-19 demonstrated that age, history of CKD, and ARF incidence affect mortality. Factors, including age and a history of CKD, affected the rate of ICU admission. Finally, a history of CKD and the incidence of ARF affected the need for invasive ventilation.

A study by Li et al., performed on 119 patients hospitalized with COVID-19 in Wuhan, China (including 123 nondiabetic patients and 76 diabetic patients), showed that the mortality rate in diabetics was significantly higher than that in the nondiabetic group (14.5% vs. 5.7%; *p* = 0.036). Among the variables of age, cardiovascular disease, CKD, hypertension, and diabetes, age and diabetes were risk factors for mortality in the whole population, similar to our results. In contrast to our study, CKD was not an influential factor in their study, which may be because of the smaller number of patients studied and/or CKD patients compared to our study.^17^

Another study conducted by Lei et al. on 288 patients hospitalized with COVID-19 in China (including 264 nondiabetic patients and 24 diabetic patients) showed that the need for ICU admission in diabetic patients was significantly higher than that in nondiabetic patients (20.8% vs. 8.3%; *p* = 0.044). The study also reported that age and diabetes were the risk factors for ICU admission in diabetic patients among different variables, including age, sex, hypertension, cardiovascular disease, and HbA1C above 7%, which was similar to our results.^18^

Ciardullo et al. conducted a study on 373 patients hospitalized with COVID-19 in Italy. They reported a mortality rate of 38% in the whole studied population, and risk factors for mortality included: age, diabetes, and COPD (chronic obstructive pulmonary disease). However, sex, hypertension, CKD, and cardiovascular disease were not contributing factors. In our study, lung disease was not an influencing factor, but CKD was an influencing factor because the number of patients with CKD in our study was almost double that in the previous study.^19^

Izzi et al.’s study performed on 889 patients hospitalized with COVID-19 in London (including 337 diabetic patients (38%)) reported that mortality and/or ICU admission occurred in 36% of all patients and among factors, including age, sex, diabetes, IHD, heart failure, hypertension, COPD, cancer, and cerebrovascular accident (CVA). It showed that the male sex and age were associated with increased risk of mortality and ICU admission (719 patients from the total population were included in the model). Mortality and/or ICU admission occurred in 44.2% of diabetic patients. In addition, age and IHD were associated with an increased risk of mortality and ICU admission in diabetic patients (268 diabetic patients were included in the analysis model). These discrepancies regarding the role of underlying diseases as effective factors in mortality and ICU admission can be attributed to differences in the frequency and race of the two populations.^20^

Acharya et al.’s study on 324 patients hospitalized with COVID-19 (16.97% diabetic) in Korea found that the mortality rate in diabetics was significantly higher than that in nondiabetics (20% vs. 4.8%). Among age, sex, hypertension, cardiovascular diseases, cerebrovascular diseases, cancer, and laboratory parameters, only age and lactate dehydrogenase were identified as risk factors for mortality in diabetics, similar to the results of our study. Acute and chronic renal failure were not examined in their study.^21^

According to a study conducted by Nicholson et al., on 1042 patients hospitalized with COVID-19 in the United States, 38.7% of patients required mechanical ventilation. Among age, sex, diabetes, cardiovascular disease, hypertension, CKD, COPD, and cancer, only diabetes was identified as an influential factor in the need for mechanical ventilation. They also reported that 20.1% of patients died in their study and that age, male gender, cardiovascular disease, and diabetes were factors contributing to mortality, which agrees with the results of our study.^22^

A study conducted by Mansour et al. at Shariati Hospital in Tehran on 353 patients admitted with COVID-19 (including 111 diabetic patients and 242 nondiabetic patients) showed no statistically significant difference between diabetic and nondiabetic patients regarding mortality rate, ICU admission and the need for mechanical ventilation. They also found no significant relationship between diabetes and mortality, and the need for ICU admission and mechanical ventilation before and after adjusting for several factors, including hypertension, IHD, cerebrovascular events, CKD, cancer, immunodeficiency, age, sex, body mass index (BMI), and smoking, which is contrary to our study results and may be because of the small number of samples studied.^23^

Patients with cardiovascular disease have higher expression of the angiotensin-converting enzyme-2 (ACE2), which could be a potential reason for their increased sensitivity to SARS-COV-2.^24^ In addition, patients with cardiovascular disease are more likely to experience blood coagulation due to elevated D-dimer levels, which increases the risk of pulmonary embolism resulting in hypoxia and heart failure.^25^ Also, in patients with acute coronary syndrome (ACS), decreased cardiac function leads to myocardial ischemia, which may be a factor in the deterioration of COVID-19 patients’ condition leading to death.^26^

By binding to the ACE2 receptor, the SARS-COV-2 virus intensifies the inflammatory phase and induces a cytokine storm that can lead to acute tubular necrosis. Meanwhile, it activates the complement system triggering cell death and damaging renal epithelial cells, and through activation of precoagulation factors, it leads to hypercoagulation. As a result of these mechanisms, ARF is created, which is an influential factor in developing severe COVID-19 disease.^27^

The present study had several limitations that are as follows: 1. The study was retrospective in nature. 2. The data were collected from only one hospital. 3. The diagnosis of diabetes was based on the previous history or taking antidiabetic drugs according to the patient's or their companion's report but not based on laboratory parameters, such as HbA1C; thus, some undiagnosed diabetics may have been included in the nondiabetic group. 4. No data were available to compare individuals with controlled and uncontrolled diabetes to show how glycemic control affected the severity of COVID-19 and mortality. 5. Data such as BMI, lipid profile, 25 (OH) Vit D level were not included in patients’ records, and their effect on disease severity and death could not be assessed. Although the sample size in our study was larger than that in most existing studies, we still recommend that future studies be conducted with a larger sample size and using the cohort method, and consider all comorbidities and laboratory parameters, such as HbA1C, BMI, lipid profile, 25 (OH) Vit D level and medications used by the patient, including antihypertensive drugs, aspirin, and antidiabetic drugs that may contribute to disease severity. Diabetics should be trained to prevent COVID-19 disease and have strict control over their blood sugar. If they contract this disease, a lower threshold for monitoring, hospitalization, and ICU transfer should be considered, and blood sugar should be closely monitored.

## Conclusion

According to the present study results, the rates of mortality, the need for ICU admission, and invasive ventilation were significantly higher among diabetic patients than nondiabetic patients. In addition, our logistic regression model for evaluating risk factors affecting mortality rate, the need for ICU admission, and invasive ventilation in diabetic patients with COVID-19 showed that age, history of CKD, and the incidence of ARF were the factors affecting mortality. In contrast, age and a history of CKD were the factors affecting the rate of ICU admission. History of CKD and the incidence of ARF were factors affecting the need for invasive ventilation. Therefore, according to the results of our study, proper screening of people at risk of diabetes, timely diagnosis and treatment of people with diabetes based on existing guidelines, accurate control of their blood sugar, and monitoring of other comorbidities, particularly renal disease, can play an essential role in the management of COVID-19 disease in diabetics.

## Acknowledgments

The present article is based on the student's dissertation, Dr. Maryam Shaabanpour Fooladi, with the registration number of D-9909 in Ahwaz Jundishapur University of Medical Sciences. This study was supported by the Vice Chancellor for Research Affairs of Ahvaz Jundishapur University of Medical Sciences.

## Figures and Tables

**Table 1:** Clinical characteristics of diabetic and nondiabetic patients hospitalized due to COVID-19.

Characteristics	Diabetics n = 325	Nondiabetics n = 325	*p* value^*^

Age (Years), mean(SD)	61.3(12.53)	52.3(17.3)	< 0.0001

Male n(%)	157(48.3)	194(59.7)	0.004

Duration of diabetes mean(y)	8.5 ± 6.5	–	–

Comorbidities n(%)			

IHD	80(26.2)	35(11.7)	< 0.0001

CVA	8(3.1)	4(1.8)	0.31

HF	9(3.4)	11(4)	0.67

HTN	174(53.5)	80(24.6)	< 0.0001

CKD	61(18.8)	25(7.7)	< 0.0001

Lung disease	21(6.5)	14(4.3)	0.22

Cancer	7(2.2)	16(4.9)	0.056

Immunosuppressive user	10(3.07)	20(6.1)	0.31

New ACS	10(3.1)	6(2.2)	0.46

New CVA	5(1.5)	1(0.3)	0.21

New HF	1(0.3)	6(1.8)	0.12

ARF	100(30.7)	67(20.6)	< 0.0001

Outcomes n(%)			

ICU admission	115(35.4)	90(27.7)	0.035

NIV	7(2.2)	11(3.4)	0.33

Invasive V.	57(17.5)	37(11.4)	0.026

Mortality	57(17.5)	39(12)	0.047


IHD: ischemic heart disease, CVA: cerebrovascular accident, HF: heart failure, HTN: hypertension, CKD: chronic kidney disease, ACS: acute coronary syndrome, ARF: acute renal failure, ICU: intensive care unit, NIV: noninvasive ventilation, Invasive V: invasive ventilation*statistically significant (*p* < 0.05)

**Table 2:** The relationship of age with mortality, the need for ICU hospitalization, and the need for invasive and NIV among diabetic and nondiabetic patients admitted to hospital with COVID-19.

Age group	Diabetes	Mortality		ICU		NIV		Invasive V.	

(years)		n (%)	PV^*^	n (%)	PV^*^	n (%)	PV^*^	n (%)	PV^*^

< 30	No	0	–	3(15.8)	0.99	0	–	0	–

	Yes	0		0		0		0	

30–39	No	7(8.8)	0.99	20(25)	0.76	3(3.8)	0.54	7(8.8)	0.65

	Yes	1(5.9)		5(29.4)		1(5.9)		2(11.8)	

40–49	No	3(5.5)	0.51	8(14.5)	0.018	2(3.6)	0.52	3(5.5)	0.036

	Yes	3(9.1)		12(36.4)		0		7(21.3)	

50–59	No	6(10.5)	0.82	13(22.8)	0.67	2(3.5)	0.99	7(12.3)	0.9

	Yes	8(9.4)		22(25.9)		2(2.4)		11(12.9)	

60–69	No	10(18.5)	0.9	20(37)	0.78	0	0.3	8(14.8)	0.67

	Yes	21(19.3)		38(34.9)		4(3.7)		19(17.4)	

70–79	No	6(18.8)	0.14	11(34.4)	0.07	4(12.15)	0.015	7(21.9)	0.92

	Yes	19(33.3)		31(54.4)		0		12(21.1)	

≥ 80	No	7(25)	0.78	15(53.6)	0.09	0	–	5(17.9)	0.51

	Yes	5(21.7)		7(30.4)		0		6(26.1)	


ICU: intensive care unit, NIV: noninvasive ventilation, Invasive V: invasive ventilation* statistically significant (*p* < 0.05)

**Table 3:** Determination of risk factors affecting mortality, need for ICU admission, and the need for invasive ventilation in all patients using logistic regression model.

Variables	Mortality			ICU admission			Invasive v.		

	PV^*^	OR	95% CI	PV^*^	OR	95% CI	PV^*^	OR	95% CI

Age	0.000	1.038	1.023–1.054	0.000	1.025	1.014–1.036	0.211	1.015	.992–1.38

Sex	0.357	1.229	0.793–1.906	0.056	1.386	.992–1.938	0.164	1.373	.879–2.145

DM	0.048	1.56	1.004–2.422	0.035	1.43	1.025–1.994	0.027	1.656	1.060–2.586

HTN	0.091	1.457	0.942–2.253	0.398	1.157	0.826–1.621	0.41	1.164	0.910–1.722

CKD	0.000	2.846	1.679–4.824	0.002	0.476	0.300–0.754	0.021	2.164	1.126–4.157

ARF	0.000	2.402	1.529–3.772	0.001	1.86	1.290–2.682	0.043	1.837	1.019–3.311

History of CVD	0.114	1.618	0.891–2.936	0.003	1.768	1.208–2.588	.997	.999	.535–1.866

New CVD	0.880	0.920	0.313–2.705	0.001	3.798	1.759–8.198	0.917	0.944	.321–2.777

Lung disease	0.171	1.774	0.781–4.031	0.719	1.141	0.556–2.340	0.057	2.1580	.978–4.765

Cancer	0.009	3.267	1.345–7.932	0.093	2.046	0.887–4.718	0.033	2.716	1.086–6.791

Immunosuppressives	0.182	1.816	0.757–4.357	0.159	1.705	0.812–3.580	0.725	.838	.313–2.247


DM: diabetes mellitus, HTN: hypertension, CKD: chronic kidney disease, ARF: acute renal failure, CVD: Cardiovascular disease, ICU: intensive care unit, Invasive V: invasive ventilation, OR: odds ratio, CI: confidence interval*statistically significant (*p* < 0.05)

**Table 4:** Determination of risk factors affecting mortality, need for ICU, and the need for invasive ventilation in diabetic patients using logistic regression model.

Variables	Mortality			ICU admission			Invasive v.		

	PV^*^	OR	95% CI	PV^*^	OR	95% CI	PV^*^	OR	95% CI

Age	0.001	1.041	1.016–1.067	0.036	1.02	1.001–1.039	0.211	1.015	0.992–1.038

Sex	0.313	1.344	0.757–2.385	0.085	1.495	0.947–2.361	0.194	1.464	0.823–2.604

HTN	0.191	1.476	0.823–2.649	0.425	1.205	0.762–1.903	0.665	1.136	0.639–2.020

CKD	0.021	2.164	1.126–4.157	0.029	1.876	1.067–3.299	0.021	2.164	1.126–4.157

ARF	0.02	2.01	1.118–3.617	0.247	1.333	0.820–2.169	0.043	1.837	1.019–3.311

History of CVD	0.114	1.618	0.891–2.936	0.236	1.345	0.823–2.196	0.997	0.999	0.535–1.866

History of CVD	0.589	0.66	0.146–2.986	0.475	1.448	0.525–3.994	0.589	0.66	0.146–2.986

Lung disease	0.851	1.114	0.360–3.445	0.788	1.133	0.455–2.819	0.437	1.514	0.531–4.317

Cancer	0.445	1.913	0.362–10.114	0.677	1.379	0.303–6.273	0.445	1.913	0.362–10.114

Immunosuppressives	0.302	2.071	0.519–8.266	0.333	1.864	0.528–6.577	0.835	1.182	0.244–5.718


HTN: hypertension, CKD: chronic kidney disease, ARF: acute renal failure, CVD: Cardiovascular disease, ICU: intensive care unit, Invasive V: invasive ventilation, OR: odds ratio, CI: confidence interval* statistically significant (*p* < 0.05)

